# EDN1 gene potentially involved in the development of acute mountain sickness

**DOI:** 10.1038/s41598-020-62379-z

**Published:** 2020-03-25

**Authors:** Jie Yu, Chuan Liu, Chen Zhang, ShiZhu Bian, Jie Yang, JiHang Zhang, XuBin Gao, YouZhu Qiu, Lan Huang

**Affiliations:** 1 Institute of Cardiovascular Diseases, Xinqiao Hospital, Army Military Medical University, Chongqing, 400037 China; 2 Department of Cardiology, Xinqiao Hospital, Army Military Medical University, Chongqing, 400037 China

**Keywords:** Cardiovascular diseases, Risk factors

## Abstract

Previous investigations have indicated that environmental and genetic factors collectively contribute to the development of acute mountain sickness (AMS), but whether the *EDN1* gene is involved in AMS remains to be elucidated. A total of 356 healthy male soldiers who had not traveled to high altitudes in the previous 12 months were enrolled in our study. All participants were taken by plane from 500 m (Chengdu in Sichuan Province) to a 3700 m highland (Lhasa) within 2 hours. Clinical data were collected within 24 hours, and pulmonary function parameters were completed simultaneously. Genotypes were obtained by using iMLDR genotyping assays. A total of 237 soldiers (66.57%) presented AMS symptoms, including headache, dizziness, gastrointestinal upset and fatigue. Soldiers with AMS showed an increase in heart rate (HR), plasma tryptophan and serotonin, and a decrease in SaO2, FEV1, PEF, FVC, V75, V50, V25 and MMF (all P < 0.01). Notably, allele T in single nucleotide polymorphism (SNP) rs2070699 showed a positive correlation with the occurrence of AMS. A general linear regression analysis showed that rs2060799, Mean Arterial Pressure (MAP), SaO2, FVC, tryptophan and serotonin were independent predictors for the occurrence of AMS. Importantly, the area under the curve (AUC) values for tryptophan (0.998), serotonin (0.912) and FVC (0.86) had diagnostic specificity and sensitivity. Our results demonstrated that AMS is accompanied by changes in lung function parameters, increased plasma tryptophan and serotonin levels, and that the EDN1 polymorphism is a potential risk factor for AMS.

## Introduction

Acute mountain sickness (AMS) is the most common form of acute altitude illness and typically occurs in unacclimated persons ascending to altitudes >2500 m^1,2^. AMS may further develop into high-altitude brain edema or high-altitude pulmonary edema, which endangers lives and reduces the working ability of populations in a highland environment; in addition, it can even cause obstacles to human migration^3,4^. Therefore, AMS has also become a public health problem to be solved.

Previous investigations have indicated that environmental and genetic factors collectively contribute to the development of AMS^5,6^. The most important environmental risk factors for the occurrence and development of AMS include the rate of elevation, the altitude at arrival, and the susceptibility of the individuals^7,8^. In addition, numerous studies have suggested that some people appear to be predisposed to developing altitude illness, suggesting a genetic contribution to susceptibility^9–12^. Genome-wide association studies and other genetic investigations have found that some genes (PPAR-alpha, EPAS1 and EGLN1) regulated through the hypoxia-inducible factor (HIF) pathway are closely related to hypoxia adaptation in native Tibetan and Andean populations at high altitudes^9–12^. More importantly, the EGLN1 gene polymorphism is associated with high-altitude adaptation and susceptibility to high altitude brain edema (HACE) in Aryan and Ayurveda populations^13,14^.

Previous work demonstrated that polymorphisms of the EPAS1 and EGLN1 genes are associated with susceptibility to acute mountain sickness (AMS) in a Han Chinese population^15–17^. More importantly, research has indicated that the EPAS1 and EGLN1 proteins interact with endothelin-1 (ET-1, encoded by the gene *EDN1*), and ET-1 variants have independent and interactive roles in the susceptibility to high altitude pulmonary edema^18^. ET-1 activation of Rho kinase (ROCK) decreases lung alveolar and vascular growth and contributes to the development of experimental bronchopulmonary dysplasia^19^. In addition, some studies suggest that ET-1 is an independent predictor of both AMS and its severity^20,21^. Therefore, the hypothesis is that the *EDN1* gene interacts with the EPAS1 and EGLN1, which together contribute to the development of AMS. Current research aims to investigate the polymorphisms of the *EDN1* gene associated with AMS in a large Han Chinese soldier population, which may contribute to the prediction of the development of AMS.

## Materials and Methods

### Literature search

A total of 356 healthy male soldiers (average age, 23.05 ± 3.99 years; height, 171.42 ± 4.96 cm; and weight, 63.86 ± 7.67 kg) who had not traveled to high altitudes in the previous 12 months were enrolled in our study according to the inclusion and exclusion criteria. Subjects with any one of the following conditions were excluded: subjects took medication or received an intervention; subjects suffered from migraines, autoimmune diseases, respiratory diseases, or cardiovascular diseases; subjects had malignancy or liver and kidney dysfunction; subjects had an active infection or a bad cold; and subjects had psychiatric disorders or neuroses that interfered with the completion of the questionnaires performed by two trained doctors. 30 peoples as a unit were recruited and informed to taken to the test site simultaneously. All subjects accepted health education and instructions so that all of them could better understand the meaning of the experimentation. A series of data such as questionnaires, symptoms, auxiliary examinations, and blood were collected and scheduled from 9:00 to 12:00 on the same day.

The participants’ baseline parameters (age, weight, height, smoking and drinking history) were measured at 500 m (Chengdu). All subjects ascended to the 3700 m highland (Lhasa) within 2 hours from 500 m (Chengdu in Sichuan Province) by plane. All participants completed the structured case report form (CRF) questionnaires, including self-reported demographic data, physiological symptoms, history of smoking and drinking and symptoms related to AMS according to the Lake Louise scoring system (LLss). Morning fasting venous blood was collected to isolate genomic DNA. Spirometric parameters were tested with a Sensor MedicsVmax229D pulmonary function instrument. Research parameters were recorded at 24 hours after the participants’ arrival at the 3700 m highland.

### Ethics statement

All procedures performed in studies involving human participants were in accordance with the ethical standards of the institutional and/or national research committee and with the 1964 Helsinki Declaration and its later amendments or comparable ethical standards. Participants who agreed to participate in the study were familiar with the purpose and process of this study and signed informed consent forms before the trial. Informed consent was obtained from all individual participants involved in the study. All study protocols were approved by the Ethics Committee of The Second Affiliated Hospital of the Army Medical University and were carried out in accordance with established national and institutional ethical guidelines regarding the involvement of human subjects and the use of humans for research. Current clinical trials were registered in Chictr.org; URL: www.chictr.org; No. ChiCTR-RCS-12002232.

### Diagnostic criteria and assessment of AMS

For diagnosis, AMS was defined according to the 2018 Lake Louise AMS score as a total of three or more points from the four symptom categories, including at least one point from headache after a recent ascent or gain in altitude^22,23^. The Lake Louise AMS questionnaire contained four different symptoms, headache, gastrointestinal symptoms, fatigue and dizziness, and each was graded from 0 to 3 in severity. The AMS consensus committee suggested no AMS as 0–2 points, mild AMS as 3–5 points, moderate AMS as 6–9 points, and severe AMS as 10–12 points^22,23^.

### Physiological data collection

After the participants arrived at 3700 m, four physiological parameters such as systolic blood pressure (BP), diastolic blood pressure (DP), serum oxygen saturation (SaO2) and heart rate (HR), were measured after resting for 30 min in a sitting position. The BP, DP and HR were measured by a sphygmomanometer (HEM-6200, OMRON, China). SaO2 was measured by a pulse oximeter (NONIN-9550, Nonin Onyx, USA). Mean artery pressure (MAP) was calculated by using the following formula: ${\rm{MAP}}=({\rm{BP}}+2\times {\rm{DP}})/3$.

### Determination of pulmonary function parameters

Pulmonary function parameters included forced expiratory volume in 1 second (FEV1), forced vital capacity (FVC) and peak expiratory flow (PEF), forced expiratory flow at 25–75% of forced vital capacity (V25, V50 and V75), and maximum mid-expiratory flow (MMF) were measured with a portable spirometer (Minato AS-507; Minato Medical Science Co., Ltd., Osaka, Japan). Each measurement was repeated twice, and the average value was used for statistical analysis. Spirometry was performed according to the guidelines of the American. Thoracic Society^24,25^. As previously reported, the flow velocity index (PEF, V75, V50, V25, MMF) for pulmonary function was corrected by air pressure using the following formula: ${\rm{Flow}}\,{\rm{rate}}={\rm{Test}}\,{\rm{flow}}\,{\rm{rate}}\times \sqrt{{\rm{Highland}}\,{\rm{atmospheric}}\,{\rm{pressure}}/{\rm{Lowland}}\,{\rm{atmospheric}}\,{\rm{pressure}}}$^26^.

### SNP selection and genotyping

A total of 3 single nucleotide polymorphisms (SNPs) within the promoter region of the *EDN1* gene were selected based on the SNP tagging of Han Chinese in Beijing from the HapMap database (http://hapmap.ncbi.nlm.nih.gov). The coverage of tagged SNPs, which was calculated by Tagger Server (http://www.broadinstitute.org/mpg/tagger/), averaged 70% of the information in the targeted region with a max r^2^ ≥ 0.8. Fasting venous blood (5 ml) was collected from each subject with EDTA-K2 anticoagulant, and the whole blood and plasma were separated and stored in a refrigerator at −80 °C. Genomic DNA was isolated from peripheral blood cells using a TIANamp Blood DNA kit (Tiangen Biotech, Beijing) according to the manufacturer’s instructions. Quality control of the DNA was conducted by agarose gel electrophoresis and λ DNA-Hind III digested bands. The SNPs (rs5370, rs2070699 and rs2248580) in the promoter region of the *EDN1* gene were genotyped in subjects, which was performed with improved multiple ligase detection reaction (iMLDR) genotyping assays with technical support from the Center for Genetic and Genomic Analysis (Genesky Biotechnologies Inc., Shanghai). The levels of tryptophan and serotonin in the plasma were measured using a high-performance liquid chromatography (HPLC) system equipped with a Shimazu RF-10 AXL fluorometer detector set at excitation and emission wavelengths of 285 nm and 345 nm, respectively.

### Statistical analysis

Clinical features are presented as the means ± standard deviations (SDs). A chi-squared test or Fisher’s exact test was used for the analysis of contingency tables depending on the sample size. Student’s t test and the Wilcoxon test were used to compare clinical characteristics between AMS and non-AMS groups by using R software (http://www.R-project.org/). Principle component analysis (PCA) was done by using R software. The Hardy–Weinberg equilibrium was calculated using theonline software PLINK. The allele and genotype distributions of the 3 SNPs within the *EDN1* gene between the AMS and non-AMS groups were calculated with PLINK (http://zzz.bwh.harvard.edu/plink/)^27^. A backward selection procedure in a stepwise regression analysis was employed to analyze the factors influencing the occurrence of AMS by using R software. Bonferroni correction in the present study minimized false positive associations to assure reliable results, although this method may have led to a relatively conservative result^28^.

## Results

### Clinical demographic features

**Figure 1:**
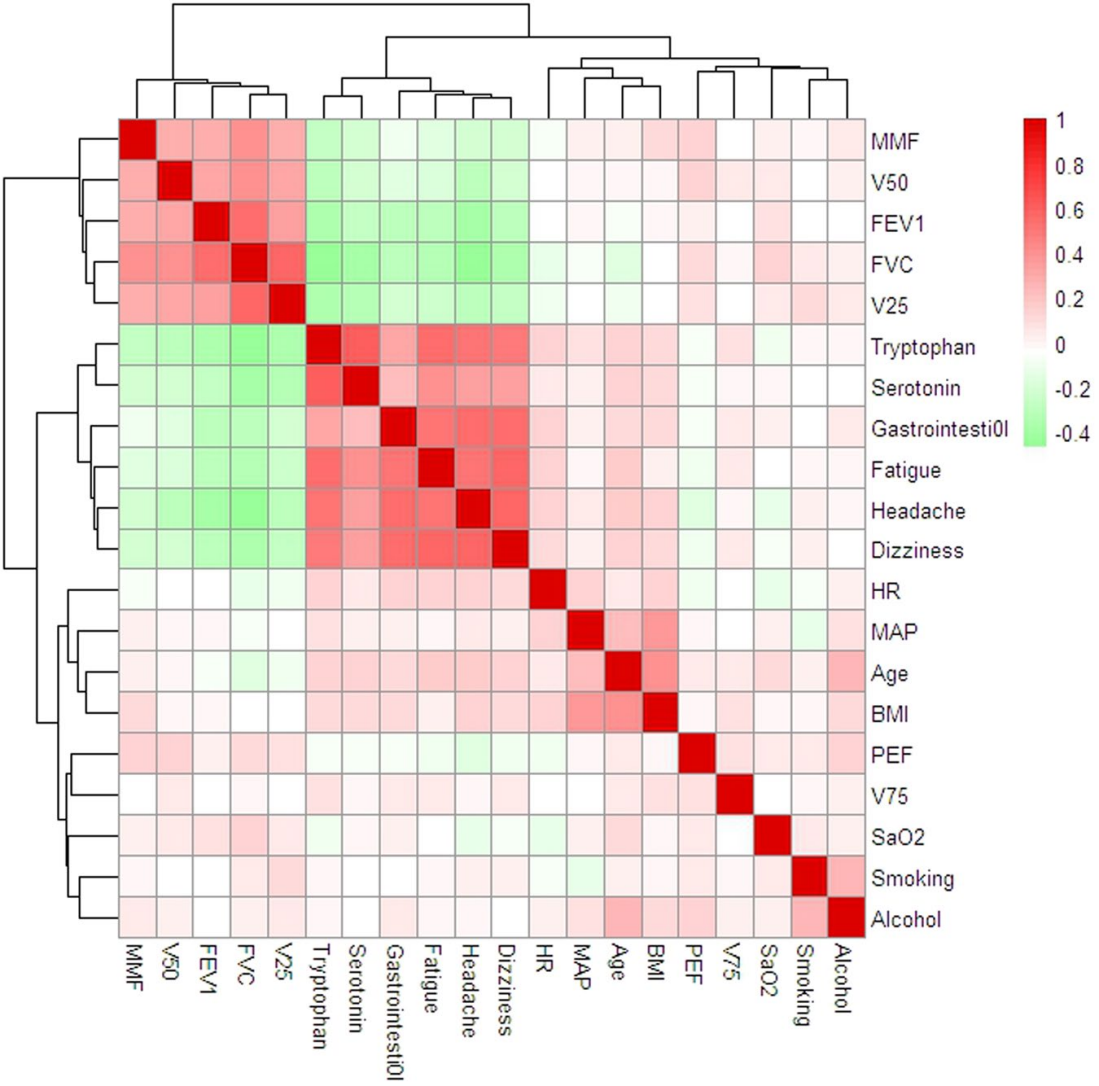
Heatmap of Pearson’s correlation between 20 clinical characteristics.

**Figure 2:**
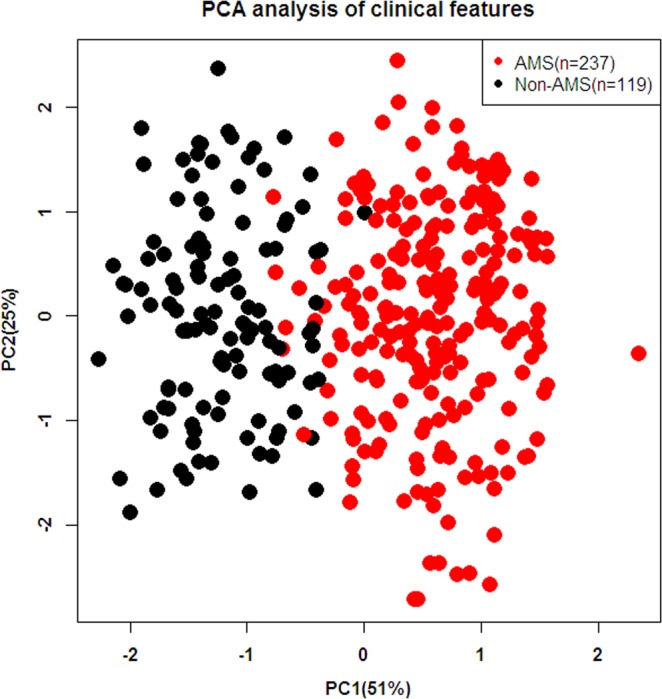
Principal components analysis (PCA) of 24 clinical features significantly distinguished the AMS group from the non-AMS group. Two main dimensions (PC1 and PC2) were used for plot.

Pearson correlation or principal component analysis (PCA) was used to determine the rationality of clinical characteristics of all participants from all participants. A total of 356 healthy male soldiers with an average age of 23.05 ± 3.99 were enrolled in the current investigation. Twenty-four clinical characteristics, including age, height, weight, and parameters of pulmonary function, were included in the current analysis. Pearson’s correlation was analyzed for these features, and the plot is presented in Fig. 1. Parameters of pulmonary function, such as V25, V50, MMF, FEV1 and FVC, were positively correlated with each other and indicated a negative correlation with AMS diagnosis. A PCA of 24 clinical features significantly distinguished the AMS group from the non-AMS group (Fig. 2).

### Comparison of clinical characteristics between the AMS and non-AMS groups

According to the diagnostic criteria and assessment of AMS, 237 soldiers were diagnosed with AMS. A comparison of clinical characteristics between soldiers with AMS and those without AMS indicated an higher in HR, tryptophan, and serotonin and a decrease in SaO_2_, FEV1, PEF, FVC, V75, V50, V25 and MMF in soldiers with AMS (Table 1). Notably, AMS symptoms were positively associated with the levels of tryptophan and serotonin, gastrointestinal symptoms, fatigue, headache, and dizziness and negatively associated with the pulmonary function levels of SaO_2_, FEV1, PEF, FVC, V75, V50, V25 and MMF (Fig. 1). Our pulmonary function results are similar to Cremona *et al*. study^29^. There were no significant differences among other features between soldiers with AMS and those without AMS (Table 1).

**Table 1:** Clinical features presentation between AMS and Non-AMS group.

	Total	AMS (n=237)	Non-AMS (n=119)	Wilcox/T*/Fisher^&^ test
Age (years old)	23.05 ± 3.99	23.44 ± 4.17	22.28 ± 3.48	0.15
BMI	21.72 ± 2.31	21.83 ± 2.42	21.50 ± 2.08	0.32
Smoking		64/1/172	34/0/85	0.87^&^
Alcohol		1/82/154	0/36/83	0.61^&^
SBP (mmHg)	117.85 ± 11.64	117.89 ± 11.78	117.77 ± 11.39	0.65
DBP (mmHg)	78.35 ± 9.89	78.39 ± 9.61	78.28 ± 10.48	0.92*
MAP (mmHg)	91.51 ± 9.99	91.54 ± 9.87	91.45 ± 10.27	0.94*
SaO2 (%)	88.15 ± 3.16	87.42 ± 3.09	89.61 ± 2.77	<0.001
HR	82.32 ± 12.49	83.7 ± 12.31	79.56 ± 12.44	<0.01*
FVC	4.00 ± 0.43	3.82 ± 0.37	4.35 ± 0.31	<0.001
FVC % predicted		88.17 ± 8.76	94.51 ± 6.82	<0.001
FEV1	3.47 ± 0.31	3.38 ± 0.29	3.67 ± 0.26	<0.001
FEV1%predicted		82.23 ± 6.03	84.94 ± 6.45	0.124
PEF	9.24 ± 0.68	9.20 ± 0.67	9.33 ± 0.70	0.09
V75	8.17 ± 0.77	8.17 ± 0.81	8.16 ± 0.89	0.72
V50	4.75 ± 0.41	4.65 ± 0.37	4.95 ± 0.42	<0.001
V25	2.16 ± 0.18	2.10 ± 0.15	2.27 ± 0.19	<0.001
MMF	4.38 ± 0.37	4.29 ± 0.36	4.56 ± 0.32	<0.001
Headache		4/161/61/11	70/49/0/0	<0.001^&^
Dizziness		11/173/46/7	72/47/0/0	<0.001^&^
Gastrointesti0l		146/67/23/1	116/3/0/0	<0.001^&^
Fatigue		4/171/51/11	82/36/1/0	<0.001^&^
Tryptophan(ng/ml)	61.49 ± 9.19	66.99 ± 4.56	50.55 ± 5.49	<0.001
Serotonin(pg/ml)	0.98 ± 0.19	1.07 ± 0.16	0.80 ± 0.09	<0.001

“/” represent different statistical method for selection. Fisher Exact Test^&^, T test* and Wilcox test. BMI, Body Mass Index; SBP, systolic blood pressure; DBP, diastolic blood pressure; MAP, Mean artery pressure; HR, heart rate; FVC, forced expiratory volume; FEV1, forced expiratory volume in 1 second; PEF, peak expiratory flow; V75, 75% vital capacity; V50, 50% vital capacity; V25, 25% vital capacity; MMF, mean maximal flow.

### Distribution of SNPs within the *EDN1* gene between the AMS and non-AMS groups

No deviation from the Hardy-Weinberg equilibrium was detected in the 3 SNPs (all P-values >0.05). Genotypic and allelic frequencies of one SNP (rs2070699) within *EDN1* showed significant differences between soldiers with AMS and those without AMS. Allele T in SNP rs2070699 (λ test, OR = 0.6652, P = 0.011) had a positive correlation with the occurrence of AMS (Table 2). Genotype TT/TG in SNP rs2070699 (Fisher’s exact test, P = 0.019) denoted a higher susceptibility to AMS (Table 2). There were no significant differences in the genotypic and allelic frequencies between any other SNPs (rs5370 and rs2248580) in the *EDN1* gene and AMS. In addition, multimarker haplotype analysis showed that 4 of the 2 haplotypes (rs3025039-rs3025040-rs10434: GGA and GTA) within the *EDN1* gene region were significantly different between soldiers with AMS and those without AMS (Globe λ test, χ^2^ = 11.85, P = 0.008, Table 3).

**Table 2:** Genotype and allele distribution of 3 SNPs within EDN1 gene between AMS and Non-AMS group.

SNP (GRCh38)	Genotype/Allele	AMS (n = 237)	Non-AMS (n = 119)	Chisq square test			Fisher P value
				λ value	OR	P value	
rs5370	TT/TG/GG	14/80/117	10/42/45				0.26
	T/G	108/314	62/132	2.67	0.73	0.1021	
rs2070699	GG/GT/TT	46/111/80	30/65/23	7.94		**0.019**	
	G/T	203/271	125/111	6.52	0.67	**0.011**	
rs2248580	CC/CA/AA	36/101/98	22/60/36				0.12
	C/A	173/297	104/132	3.32	0.74	0.068

**Table 3:** Multimarker haplotype (rs5370-rs2070699-rs2248580) analysis within the *EDN1* gene region in AMS and Non-AMS subjects.

Haplotype	Frequency in AMS	Frequency in Non-AMS	χ^2^ (1 df)	P (adjust)
TGC	0.26	0.31	2.30	0.13
GGC	0.11	0.11	7.1 × 10^−7^	1.00
GGA	0.06	0.12	7.09	0.01
GTA	0.57	0.46	7.81	0.005

Globe χ^2^ = 11.85; df = 3; P = 0.008.

### Association of the SNP rs2070699 genotype with clinical characteristics

**Figure 3:**
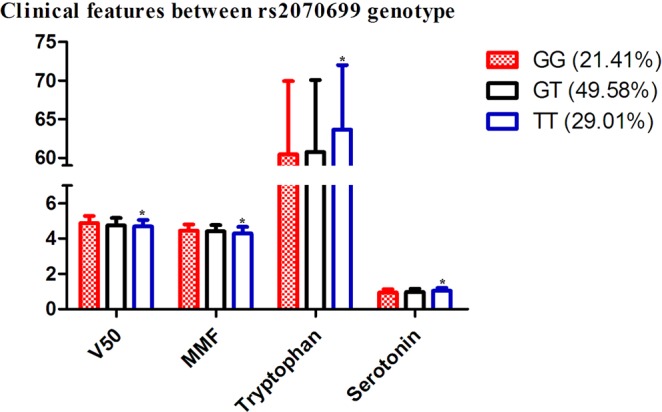
Four clinical characteristic distributions in the SNP rs2070699 genotype.

Four clinical features (V50, MMF, tryptophan and serotonin) showed different distributions between SNP rs2070699 genotypes (Fig. 3). rs2070699 TT genotype showed higher tryptophan and serotonin level and lower V50 and MME value compared with GG genotype (Wald test, P < 0.05, Fig. 3). The regression coefficients of SNP rs2070699 with V50 and MMF were 0.0912 and 0.07936, respectively, which denoted that genotype GG endured higher V50 (Wald test T = 2.974, P = 0.0031, Table 4) and MMF (Wald test T = 2.9, P = 0.00397, Table 4) scores compared to genotype TT. Conversely, the regression coefficient of SNP rs2070699 with tryptophan and serotonin was −1.703 and −0.04891, which indicated a higher level of tryptophan and serotonin in individuals with genotype TT.

**Table 4:** Association of SNP rs2070699 genotype with clinical features.

	Regression coefficient (β)	Standard error	Regression R^2^	Wald test T	Wald test Pvalue
V50	0.09	0.03	0.02	2.97	0.003
MMF	0.08	0.03	0.02	2.90	0.004
Tryptophan	−1.70	0.69	0.02	−2.48	0.01
Serotonin	−0.05	0.014	0.03	−3.52	0.0005

V50, 50% vital capacity; MMF, mean maximal flow. All parameter were collected in high altitude.

### General linear regression analysis of influencing factors in AMS

**Figure 4:**
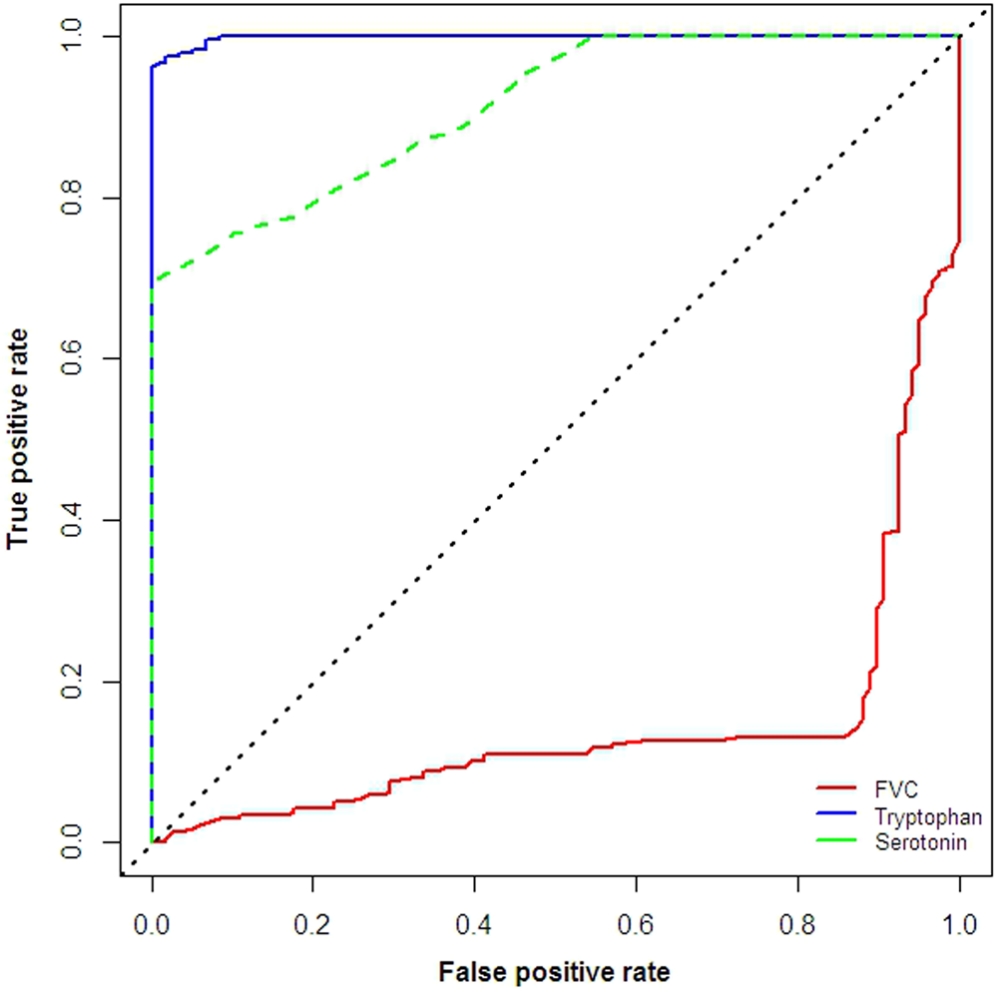
The ROC curve of FVC, tryptophan and serotonin for the occurrence of AMS.

In the general linear regression analysis, only rs2060799 (t = 2.581, P = 0.0136), MAP (t = −1.987, P = 0.04767), SaO_2_ (t = −3.317, P = 0.00101), FVC (t = −6.794, P < 0.001), tryptophan (t = 18.305, P < 0.001) and serotonin (t = 6.369, P < 0.001) remained independent predictors of AMS (Table 5). Figure 4 shows the ROC curve of FVC, tryptophan and serotonin for the AMS group. Importantly, the area under the curve (AUC) for tryptophan (0.998) was significantly greater than those of serotonin (0.912, p < 0.01 for AUC comparison) and FVC (0.86, p < 0.01 for AUC comparison). Simultaneously, the area of the ROC curve for serotonin (0.912) was significantly greater than that of FVC (0.86, p < 0.001 for AUC comparison).

**Table 5:** Stepwise backward linear regression analysis of AMS in 356 patients.

	Estimate	Std.Error	t.value	Pr(>|t|)
(Intercept)	1.33	0.42	3.15	**0.002**
Age	0.003	0.003	0.78	0.44
BMI	−0.003	0.006	−0.44	0.66
Smoking	0.02	0.014	1.61	0.11
Alcohol	−0.02	0.013	−1.47	0.14
rs2070699	0.10	0.03	2.58	**0.014**
MAP	−0.003	0.001	−1.99	**0.048**
SaO_2_	−0.013	0.004	−3.32	**0.001**
HR	0.0013	0.001	1.28	0.20
FVC	−0.21	0.03	−6.79	**<0.001**
Tryptophan	0.03	0.002	18.31	**<0.001**
Serotonin	0.51	0.079	6.37	**<0.001**

BMI, Body Mass Index; MAP, Mean artery pressure; HR, heart rate; FVC, forced expiratory volume.

## Discussion

In the current study, 356 healthy male soldiers endured a sharp ascent from 500 m to 3700 m within 2 hours. In this process, 237 soldiers (66.57%) endured AMS symptoms, including headache, dizziness, gastrointestinal upset and fatigue. Soldiers with AMS showed an increase in HR, tryptophan, and serotonin and a decrease in SaO_2_, FEV1, PEF, FVC, V75, V50, V25 and MMF. More importantly, allele T in SNP rs2070699 denoted a positive effect on AMS, and general linear regression analysis revealed that rs2060799, MAP, SaO_2_, FVC, tryptophan and serotonin remained independent predictors of AMS. To our knowledge, this is the first study to investigate the factors influencing the occurrence of AMS in a Han Chinese population.

The standardized diagnostic clinical criteria for AMS were determined by the Lake Louise scoring system (LLss), which uses several symptoms for clinical diagnostic criteria. The Lake Louise score was developed at the International Hypoxia Conference in Lake Louise and is an internationally accepted standard for the diagnosis of AMS. AMS is characterized by nonspecific symptoms and distinct physiological outcomes. The main symptoms of AMS are headache, anorexia, nausea, vomiting, fatigue and dizziness, but not all will be present at the same time. Headache is an essential symptom for the diagnosis of AMS. The symptoms of AMS generally occur 6 to 12 hours after arrival at high altitudes. The LLss mainly employs four symptoms for scoring including headache, dizziness, gastrointestinal reactions and fatigue. After reaching high altitude, those with a score greater than or equal to 3 points and an accompanying headache received a diagnosis of AMS. If patients had a score of greater than or equal to 3 points and less than 5 points, they were diagnosed with mild AMS. Similarly, a score of greater than or equal to 5 points was recognized as severe AMS. According to the criteria, a previous investigation indicated that approximately 10% of individuals will suffer from AMS at 2500 m, and the incidence of AMS will exceed 60% at 4500 m^28^. In our primary reports, 66.57% of soldiers endured AMS after sharply ascending to a highland within 2 hours, which was a higher incidence than reported in other investigations. Therefore, investigating the potential factors that influence AMS is necessary.

The range of elevation is the main factor for AMS^30^. Previous studies have shown that acute highland disease generally occurs after a person rises to 2500 meters above sea level, and its incidence and severity increase with altitude. At an altitude of 2500 meters, the incidence of acute high-altitude disease is approximately 10% to 25%, and symptoms are usually mild. However, at 4500 to 5500 m, the incidence of acute high-altitude sickness is between 50% and 85%^8,31^. A retrospective study showed that the main independent risk factors for acute high-altitude disease include a history of acute high-altitude disease, a rapid rise (in altitudes over 2000 meters, rising altitudes ≥ 625 meters per day), and a lack of previous exercise^32^. Other possible risk factors include female sex, age under 46 years, and a history of migraines. Exercise may aggravate acute high-altitude disease, but good physical fitness is not a protective factor. A meta-analysis by Vinnikov D showed that smoking may also be a risk factor^33^, but there was no significant difference in body mass index, age, or smoking and drinking status between the AMS group and the non-AMS group after high-altitude exposure, indicating that our sample homogeneity was relatively good. However, our current report indicated that the rs2070699 polymorphism within the *EDN1* gene was an independent predictor of AMS, which was notable.

Current reports have demonstrated that rs2070699 of the *EDN1* gene is a possible susceptibility factor that influences AMS. The frequency of the T allele of rs2070699 in the *EDN1* gene is 0.545 from the 1000 genome database. Data from current research indicated that the T allele frequency was 0.5365 (191/365), which denotes no differential distribution. However, in the AMS group, the T allele frequency was 0.571 (271/474) and was highly enriched in the AMS group (Poisson distribution test, P < 0.05). The mechanism behind *EDN1* and the occurrence of AMS still needs further investigation. Previous studies may offer some clues about the connection between *EDN1* and AMS. Serotonin is an important physiologically active substance in the body. The precursor of serotonin is tryptophan. Tryptophan becomes serotonin under the catalysis of enzymes in the body. Serotonin has a strong contractile vasoconstriction in the lungs as well as in peripheral blood vessels. In addition, serotonin can increase the vasoconstrictor effects of other vasoconstrictors (angiotensin I, norepinephrine, ET-1)^34,35^. Determination of serum tryptophan and serotonin concentration can indirectly reflect the body’s vasoconstriction. Research suggests ET-1-ROCK interactions have already been proven to contribute to decreased alveolar and vascular growth and pulmonary hypertension (PH) in experimental bronchopulmonary dysplasia^19,36^ confirmed that ET-1-mediated positive inotropic effects and myocardial fetal gene induction further lead to PH. Interestingly, recent reports from Josefa *et al*. demonstrated that melatonin, which is an indoleamine that is synthesized from tryptophan under the control of the enzymes aryl alkylamine N-acetyltransferase (AANAT) and acetyl serotonin methyltransferase (ASMT), can reduce endothelin-1 expression through the inactivation of FoxO-1 and NF-κβ, playing an important role in colon cancer^37^. Coincidently, our results show that tryptophan and its metabolite product serotonin are more highly expressed in the AMS group than in the non-AMS group. Therefore, tryptophan and its metabolism may influence the expression of EDN1 through the inactivation of FoxO-1 and NF-κβ expression, which further leads to AMS. However, whether tryptophan and serotonin regulate the expression of *EDN1* requires further validation.

Two limitations in the current report must be mentioned. First, only select SNPs of the *EDN1* gene were considered in a relatively small sample, which may influence the effect size in the association study. Second, the current study did not perform a functional experiment for the rs2070699 genotype in the expression of the *EDN1* protein; therefore, direct evidence to show that rs2070699 regulate the expression of *EDN1* is lacking.

In summary, our results demonstrated that AMS is accompanied by changes in lung function parameters, increased plasma tryptophan and serotonin levels, and that the EDN1 polymorphism is a potential risk factor for AMS.
